# Infliximab therapy in rheumatoid arthritis and ankylosing spondylitis-induced specific antinuclear and antiphospholipid autoantibodies without autoimmune clinical manifestations: a two-year prospective study

**DOI:** 10.1186/ar1440

**Published:** 2004-09-23

**Authors:** Carole Ferraro-Peyret, Fabienne Coury, Jacques G Tebib, Jacques Bienvenu, Nicole Fabien

**Affiliations:** 1 UF Autoimmunité, Laboratoire d'Immunologie, Centre Hospitalier Lyon-Sud, Pierre Bénite, France; 2 Service de Rhumatologie, Centre Hospitalier Lyon-Sud, Pierre Bénite, France

**Keywords:** ankylosing spondylitis, anti-β_2_-glycoprotein I autoantibodies, antiphospholipid autoantibodies, infliximab, rheumatoid arthritis.

## Abstract

Treatment of rheumatoid arthritis (RA) with infliximab (Remicade^®^) has been associated with the induction of antinuclear autoantibodies (ANA) and anti-double-stranded DNA (anti-dsDNA) autoantibodies. In the present study we investigated the humoral immune response induced by infliximab against organ-specific or non-organ-specific antigens not only in RA patients but also in patients with ankylosing spondylitis (AS) during a two-year followup. The association between the presence of autoantibodies and clinical manifestations was then examined. The occurrence of the various autoantibodies was analyzed in 24 RA and 15 AS patients all treated with infliximab and in 30 RA patients receiving methotrexate but not infliximab, using the appropriate methods of detection. Infliximab led to a significant induction of ANA and anti-dsDNA autoantibodies in 86.7% and 57% of RA patients and in 85% and 31% of AS patients, respectively. The incidence of antiphospholipid (aPL) autoantibodies was significantly higher in both RA patients (21%) and AS patients (27%) than in the control group. Most anti-dsDNA and aPL autoantibodies were of IgM isotype and were not associated with infusion side effects, lupus-like manifestations or infectious disease. No other autoantibodies were shown to be induced by the treatment. Our results confirmed the occurrence of ANA and anti-dsDNA autoantibodies and demonstrated that the induction of ANA, anti-dsDNA and aPL autoantibodies is related to infliximab treatment in both RA and AS, with no significant relationship to clinical manifestations.

## Introduction

Clinical trials in rheumatoid arthritis (RA) have demonstrated that antibodies directed against tumor necrosis factor α(TNF-α) (adalimumab, infliximab [Remicade^®^]) are highly beneficial for most patients who are refractory to classic treatment with disease-modifying anti-rheumatic drugs, methotrexate or steroid therapy [1-4]. These anti-inflammatory effects of infliximab have led to their use in other inflammatory diseases such as Crohn's disease [5] and ankylosing spondylitis (AS), with a similar efficacy to that in RA [6-8].

The side effects of these treatments are acknowledged to be very infrequent, with the exception of opportunistic intracellular infection, due particularly to the reactivation of latent *Mycobacterium tuberculosis*. The other major side effects are an exacerbation of demyelinating disorders and the induction of severe neutropenia and thrombocytopenia [1,2,4,9-11]. Infusion reactions have also been observed and have been correlated with the induction of anti-chimeric antibodies against infliximab [12]. The development of autoantibodies that are usually associated with systemic lupus erythematosus (SLE), namely antinuclear (ANA) and anti-double-stranded DNA (anti-dsDNA) autoantibodies, has also been observed after infliximab treatment in 63.8% and 13% of RA patients and in 49.1% and 21.5% of Crohn's disease patients, respectively [13-15]. Among the sera that were positive for anti-dsDNA autoantibodies, 9% were also positive for anti-Sm autoantibodies, which are specific for SLE [13]. However, only a few cases of SLE-like syndrome have been reported in infliximab-treated patients [9,13,16-18].

As yet, the occurrence of other autoantibodies has not been clearly demonstrated, such as antiphospholipid (aPL) autoantibodies and anti-β_2_-glycoprotein I (anti-β_2_GPI) autoantibodies, which are often associated with SLE [19,20], or autoantibodies associated with vasculitis, autoimmune hepatitis or autoimmune endocrine diseases, which have been reported in therapy that interferes with cytokine balance [21].

In the present study we investigate the prevalence of such autoantibodies during 2 years of follow-up in patients with RA or AS successfully treated with infliximab. The aim of the study was to discover whether the humoral response induced by infliximab is restricted to non-organ specific autoantibodies and to identify any associated clinical presentations, with the aim of monitoring their occurrence by detecting these autoantibodies. Concurrently, 30 patients whose RA was controlled only by methotrexate were analyzed at 1-year intervals as controls for autoantibody production.

## Materials and methods

### Patient sera

Twenty-four patients with RA and 15 patients with AS, fulfilling the ACR criteria [22] and the modified New York criteria [23], respectively, were monitored for autoantibody production over a 2-year period during which they were good responders, as defined by the modified disease activity scores [24], to a combination of methotrexate and infliximab. Concurrently, 30 RA patients well controlled by methotrexate for 6–15 years (mean 12 years) gave blood samples at 1-year intervals as controls for autoantibody production. Demographic and clinical statuses are presented in Table 1. Patients were followed clinically by the same physician during this period at regular intervals and in particular when they were receiving infliximab infusions. Clinical assessment (painful and swollen joint count, spine stiffness, careful examination of side effects, significant concomitant clinical features suggestive of infections or autoimmune disorders) were recorded accurately (Table 1). Nine patients discontinued infliximab treatment before the end of the study, between 3 and 18 months, because of adverse events, treatment inefficacy or severe infectious disease. Further details are given in Table 1.

### Treatment protocol

Twenty-four RA and 15 AS patients were treated with infliximab (Centocor, Malvern, PA, USA). In RA patients, infliximab was administered in accordance with the schedule of the ATTRACT phase III clinical trials [4]. Patients were given infliximab at a dose of 3 mg/kg at 0, 2, 4 and 6 weeks and thereafter every 8 weeks. In AS patients, after the initial 6-week protocol with 5 mg/kg, infliximab was delivered every 6 or 8 weeks, depending on the clinical response. When AS patients presented a remission, the timing of infusions was dictated by disease relapse [25].

### Follow-up of autoantibodies

Tests for autoantibodies were performed at baseline before the start of infliximab treatment and during the 24-month duration of infliximab treatment as indicated below. The sera of the 30 control RA patients were analyzed twice with a 1-year interval.

### Detection of ANA

Tests for ANA were performed at the start of infliximab treatment and at 6, 12, 18 and 24 months, by an indirect immunofluorescence technique (IIF) using HEp2 cells (Bio-Rad, Marnes-la-Coquette, France). Sera were diluted 1:80 and the conjugate was a goat anti-human F(ab')_2 _IgG, A, M (H+L) antibody conjugated to fluorescein isothiocyanate (diluted 1:100) (Bio-Rad). Classic titration of each ANA positive at a titer of 1:80 was performed by serial dilutions to 1:5120. A titer equal to or greater than 1:160 was interpreted as a positive result. For positive sera that had nuclear granular or cytoplasmic staining, the identification of autoantibodies against (ENA) was further investigated by enzyme-linked immunosorbent assay (ELISA) with an anti-human IgG (H+L) conjugate (Biomedical Diagnostics, Marne-la-Vallée, France).

### Detection of anti-dsDNA autoantibodies

Tests for anti-dsDNA autoantibodies were performed at the start of infliximab treatment and, depending on the formation of ANA, at 6, 12, 18 and 24 months of treatment with the use of a radioimmunological test (Dade Behring, Paris, France) in accordance with the manufacturer's instructions. A titer equal or greater than 5 IU/ml was interpreted as a positive result. For positive sera, the anti-dsDNA autoantibody isotype was determined by ELISA (Pharmacia, Freiburg, Germany) with an anti-human IgG (H+L) (Bio-Rad) or an anti-human IgM (H+L) (Dako) conjugate.

### Detection of anti-smooth muscle (SMA), anti-mitochondrial (AMA), anti-liver kidney microsomes (LKM), anti-thyroid peroxidase (TPO), anti-thyroglobulin (TG) and anti-adrenal (ADA) autoantibodies

Tests for SMA, AMA, LKM, TPO, TG and ADA autoantibodies were performed at the start of infliximab treatment and then at 3, 6, 12, 18 and 24 months. The sera of the 30 control RA patients were analyzed twice with a 1-year interval. For SMA, AMA, LKM and ADA, sera diluted 1:30 were tested on mouse stomach, kidney, liver or adrenal sections (Biomedical Diagnostics), with the same technique as described for ANA. For TPO and TG autoantibodies, an ELISA technique was performed in accordance with the manufacturer's instructions with an anti-human IgG (H+L) conjugate (Pharmacia, Saint-Quentin-en-Yvelines, France).

### Detection of anti-neutrophil cytoplasmic autoantibodies (ANCA)

Tests for ANCA were performed at the start of infliximab treatment and then after 6 and 12 months. The sera of the 30 control RA patients were analyzed twice with a 1-year interval. The sera diluted 1:20 were tested by IIF on human neutrophils fixed in ethanol (Menarini Diagnostics, Antony, France) with the same technique as for ANA. Positive sera were further tested for reactivity against myeloperoxidase and proteinase 3 by using an ELISA with an anti-human IgG (H+L) conjugate (Bioadvance, Emerainville, France). Titers were considered positive when they were 20 arbitrary units (AU)/ml or more.

### Detection of aPL and anti-β_2_GPI autoantibodies

Investigation of aPL autoantibodies was performed by the detection of anticardiolipin autoantibodies (ACL). ACL and anti-β_2_GPI autoantibodies were evaluated at baseline and at 6, 12 and 24 months after the start of infliximab treatment. The sera of the 30 control RA patients were analyzed twice with a 1-year interval. ACL were detected with an ELISA in accordance with the manufacturer's instructions by using anti-human IgG (H+L) or IgM (H+L) conjugates. Values were expressed as arbitrary G phospholipid (GPL) or M phospholipid (MPL) units. Positive results were graded as low positivity (IgG 11–23 GPL, IgM 6–10 MPL), moderate positivity (IgG 24–39 GPL, IgM 11–29 MPL) or high positivity (IgG ≥ 40 GPL, IgM ≥ 30 MPL). The anti-β_2_GPI autoantibodies were detected by using a home-made assay previously described [26] with serum samples diluted 1:50 and peroxidase-conjugated anti-human IgG (H+L) or IgM (H+L) (Cappell, ICN Biomedicals, OH, USA) diluted 1:400. Positive results were graded as low positivity for a value from a ratio of 1.2–1.9 AU/ml (attenuance ['optical density'] of the sample divided by attenuance of the cut-off), moderate positive for a value of ratio between 2 and 3 AU/ml and high positive for a value superior to a ratio of 3 AU/ml. The cut-off was determined by using the mean plus 5 standard deviations of attenuance of 100 sera from blood donors (data not shown).

### Statistics

Statistical analysis (95% and 99% confidence interval) was performed with the χ^2 ^test when applicable and with Fisher's exact test in other conditions.

### Ethics

Written informed consent was obtained from all patients and the study was approved by the Research and Ethics Committee of the Hospices Civils de Lyon.

## Results

### Occurrence of ANA and anti-dsDNA autoantibodies in RA and AS patients

At baseline, 9 of 24 (37.5%) infliximab-treated RA patients, 2 of 15 (13.3%) AS patients and 5 of 30 (16.7%) control RA patients were tested positive for ANA (Table 2). After 12 months of therapy, the induction of ANA was observed in 12 infliximab-treated RA patients, 8 AS patients and 4 control RA patients. At that time, the total number of positive ANA patients was 21 of 24 (87.5%) for infliximab-treated RA patients, 10 of 15 (66.7%) AS patients and 9 of 30 (30%) control RA patients. The difference between the number of induced ANA compared with the number of positive ANA at baseline was statistically significant (*P *< 0.0001) for infliximab-treated RA and AS patients, whereas the difference was not significant for the RA control group. The difference in induction was also significant for the infliximab-treated RA patients (*P *< 0.0001) comparing the two RA groups.

After 2 years of infliximab therapy, ANA became positive in one other infliximab-treated RA patient and three more AS patients, giving a total induction of 87% in RA and 85% in AS. The induction of ANA appeared between 3 and 18 months (mean 6.35 months) for RA and between 3 and 24 months (mean 10.6 months) for AS. Except in two RA patients, all the induced ANA were still positive at the end of the study, including in eight of nine patients who discontinued the treatment. One RA became negative 3 months after the end of the treatment. Furthermore, in six of the nine sera of infliximab-treated RA patients positive at baseline, the ANA titer increased up to twofold (data not shown). The titer of ANA showed a higher level between the positive ANA at the baseline compared with the titer of induced ANA, but the difference was not significant. In most ANA-positive sera during infliximab treatment, the pattern of staining was homogeneous. Two of the 22 infliximab-treated RA ANA-positive sera had granular nuclear staining characteristic of ENA. The specific target could not be identified with the use of the classic ELISA kit for ENA detection.

For anti-dsDNA autoantibodies, 1 of 24 infliximab-treated RA patients (4.2%), 2 of 15 AS patients (13.3%) and none of the control RA patients were positive at baseline (Table 2). After 12 months of treatment, induction of anti-dsDNA autoantibodies was observed in 10 of 23 (46.5%) infliximab-treated RA patients, 3 of 13 (23%) AS patients and 2 of 30 (6.7%) control patients. The induction was observed between 3 and 12 months. Three further infliximab-treated RA patients and one further AS patient became positive at 18 and 24 months, respectively, giving a total induction of 57% in RA and 31% in AS.

After the 2-year follow-up, the total number of positive patients was 14 of 24 (58.33%) for infliximab-treated RA patients, 6 of 15 (40%) for AS patients and 2 of 30 (6.7%) for control RA patients. All patients who became positive for anti-dsDNA autoantibodies were also positive for ANA. All the induced anti-dsDNA autoantibodies remained positive until the end of the study, including in three of four positive patients who discontinued the treatment. One RA patient became negative 3 months after the end of the treatment. The difference between the number of induced anti-dsDNA autoantibodies and the number of positive anti-dsDNA autoantibodies at baseline was statistically significant for the infliximab-treated RA patients (*P *< 0.0001) and for the infliximab-treated AS patients (*P *< 0.02) compared with the RA control group. Comparing the two RA groups, the difference in induction was also significant for the infliximab-treated RA patients (*P *< 0.0001). The titer of anti-dsDNA autoantibodies showed a higher level between the positive autoantibodies at baseline compared with the induced autoantibodies. The formation of ANA and anti-dsDNA autoantibodies was not linked to clinical events, namely infectious side effects, allergy or lack of efficacy.

### Occurrence of aPL/ACL and anti-β_2_GPI autoantibodies in RA and AS patients

At baseline, no RA or AS patients were positive for ACL or for anti-β_2_GPI autoantibodies. At the end of the study, significant levels of ACL were found in infliximab-treated RA patients (5 of 24, *P *< 0.01) and in infliximab-treated AS patients (4 of 15, *P *< 0.01) compared with the RA control group (Table 3).

Induction of anti-β_2_GPI autoantibodies was observed in 2 of 24 infliximab-treated RA patients and in none of the control RA patients (Table 3). The difference was not significant between the two RA populations nor within the RA and AS group, comparing the number of induced autoantibodies at baseline and after treatment. In infliximab-treated RA patients, sera were positive for ACL or anti-β_2_GPI autoantibodies (Table 3). In the group of AS patients, the two induced sera were positive for both ACL and anti-β_2_GPI autoantibodies.

All ACL and anti-β_2_GPI autoantibodies were from patients positive for ANA. Five of 11 ACL or anti-β_2_GPI autoantibody-positive sera were positive for anti-dsDNA autoantibodies. Induction did not occur simultaneously and did not seem to be determined by clinical events. Two AS sera positive for anti-dsDNA autoantibodies at baseline became positive for ACL autoantibodies 6 and 10 months after the beginning of treatment. For the other sera, anti-dsDNA, ACL and anti-β_2_GPI autoantibodies developed between 6 and 12 months after the start of treatment. No correlation was found between the occurrence of side effects (including infections), clinical status (including lupus-like symptoms, thrombopenia or thrombosis) and anti-β_2_GPI or ACL autoantibodies.

### Isotypes of induced anti-dsDNA, aPL/ACL and anti-β_2_GPI autoantibodies in RA and AS patients

Most of the anti-dsDNA autoantibodies detected during infliximab treatment of RA patients, of AS patients and in the control RA population were of IgM isotype (11 of 13 [85%], 4 of 4 [100%] and 2 of 2 [100%] respectively). One of the sera from infliximab-treated RA patients and one from AS patients were positive for both IgG and IgM. Two sera in the infliximab-treated RA group were positive only for IgG. The presence of the IgG isotype was not associated with any particular clinical pattern such as infections, lupus-like syndrome or side effects of infusion.

Among the ACL, five of five infliximab-treated RA patients and one of four AS patients were of IgM isotype. Three AS patients were of IgG isotype. The isotypes of the positive anti-β_2_GPI autoantibodies were IgM and IgG (one case), IgG (one case) for RA and IgM or IgG for AS. As for the IgG isotype in induced anti-dsDNA autoantibodies, no significant clinical association was observed in patients presenting the IgG ACL and/or anti-β_2_GPI autoantibody profile.

### Occurrence of TPO, TG, AMA, LKM, SMA, ADA and ANCA autoantibodies

Three of the 24 (12.5%) infliximab-treated RA patients, 6 of 30 (20%) control RA patients and no AS patients had TPO or TG autoantibodies at baseline. Patients with RA remained positive during infliximab treatment and at the 1-year intervals of methotrexate treatment. Only one patient (1 of 21, 4.8%) in the infliximab-treated RA group developed both TPO and TG autoantibody positivity after 12 months of treatment.

One of 24 infliximab-treated RA patients (4.2%) and 2 of 15 AS patients (13.3%) who were negative at baseline became positive for ANCA as determined by IIF. The target of these ANCA was identified by ELISA as proteinase 3 for two sera (25 and 40 AU/ml) and both myeloperoxidase and proteinase 3 for one serum (45 and 30 AU/ml). For the RA control group, ANCA were observed in two patients at baseline and remained positive at 1 year. The target of these ANCA was neither proteinase 3 nor myeloperoxidase. No other patient developed such autoantibodies after the 1-year interval analysis.

Three of the 24 infliximab-treated RA patients (12.5%) and 2 of 30 RA controls (6.7%) were SMA positive at baseline. Three of 21 infliximab-treated RA sera (14.3%) and 2 of 15 AS sera (13.3%) that were negative at baseline became positive for SMA autoantibodies at 1.5, 3, 6, 3 and 6 months respectively. These SMA autoantibodies were not antiactin autoantibodies, the only autoantibodies that are specific for autoimmune hepatitis.

Neither RA nor AS patients developed AMA, LKM or ADA autoantibodies.

The occurrence of TPO, TG, ANCA, AMA, LKM, SMA or ADA autoantibodies during infliximab therapy was not statistically significant.

## Discussion

The occurrence of a large panel of autoantibodies that are considered as biological markers of various autoimmune diseases has been investigated in a population of RA and AS patients treated with infliximab for 2 years, the longest period described so far for this kind of management. To avoid the bias of spontaneous autoantibody production under methotrexate, a control population of RA patients treated only with methotrexate was analyzed in parallel at 1-year intervals.

ANA, anti-dsDNA and aPL were the only autoantibodies to be significantly induced by infliximab treatment in RA and AS patients. This induction has already been described for ANA and anti-dsDNA autoantibodies [13,27] but our study demonstrates for the first time that infliximab treatment can also induce aPL autoantibodies in both RA and AS patients.

Our observation of ANA in up to 91.7% and 86.7% of RA and AS patients, respectively, after infliximab therapy is consistent with recent data published during the course of the present study [27]. However, the occurrence of anti-dsDNA autoantibodies was higher in our study for both RA and AS [27,28]. These discrepant results may be due to the longer period of our analysis. Indeed, the previous study analyzed this occurrence for 8.5 months after the initiation of infliximab treatment [27]; we found that anti-dsDNA autoantibodies can be induced after this period, the latest induction being found 24 months after the onset of infliximab treatment.

Clinical monitoring of the patients did not show any symptoms characteristic of SLE in the subgroup that was positive for ANA/anti-dsDNA autoantibodies.

Antiphospholipid autoantibodies were induced in 21% (5 of 24) and 27% (4 of 15) of our RA and AS patients, respectively. Anti-β_2_GPI autoantibodies were induced in 8% and 13% of our RA and AS patients, respectively. Until now, the induction of such autoantibodies has not been described in patients treated with infliximab therapy. However, it has been demonstrated in a single study in 5 of 8 (63%) RA patients treated with etanercept [29]. In that study, the presence of aPL autoantibodies along with anti-dsDNA autoantibodies was concomitant with several infections [29]. In our study, we also found an association between anti-dsDNA and aPL autoantibodies but clinical monitoring of the patients did not show any relationship between a particular serological profile and the occurrence of infection, thrombosis or thrombocytopenia for the aPL autoantibody-positive subgroup.

In contrast with other studies showing aPL autoantibodies in RA and AS populations, we found no aPL autoantibodies at baseline [30-32]. Most of the studies reporting a high frequency of aPL autoantibodies were conducted with non-standard tests; furthermore, the titers of most of the positive sera were very low. The difference in sensitivity might also be due to the choice of a different cut-off of positivity for the aPL autoantibody test and to the different clinical characteristics of the patients analyzed. Thus, the ACL test is not specific with low-positive results, so we chose a high cut-off for this test [33].

The induction of ANA and aPL autoantibodies was clearly due to infliximab, especially in the RA group, because no such induction was observed in the control RA group treated with methotrexate alone. The mechanisms that underlie autoantibody development during infliximab treatment are intriguing. These autoantibodies do in fact occur in a variety of disorders, such as RA, AS and Crohn's disease, which are characterized by different physiopathological mechanisms and different doses of infliximab. One can then postulate that this particular induction is due to the partial blockage of TNF-α induced by infliximab therapies. The role of the disturbance of the cytokine network in such induction has already been demonstrated for another cytokine, interferon γ, inducing the development of autoantibodies in patients with hepatitis C viral infection or RA [21,34,35].

Induction of autoantibodies could be a predictable consequence of anti-TNF-α blockade because this blockade could promote humoral autoimmunity by inhibiting the induction of cytotoxic T lymphocyte response, which normally suppresses autoreactive B-cells [36]. Infliximab might also act by neutralizing the biological activity of TNF-α by binding the soluble forms of TNF-α, thereby preventing the interaction of TNF-α with its cellular receptors, p55 and p75. Infliximab also binds the transmembrane form of TNF-α and could induce antibody-dependent or complement-dependent cellular cytotoxicity of the cells expressing the cytokine [37]. Furthermore, infliximab has been shown to increase the number of apoptotic T lymphocytes in the lamina propria [38] and apoptotic monocytes in peripheral blood in Crohn's disease [39]. In this case, one hypothesis concerning the development of autoimmune diseases such as SLE is that an increased apoptotic process could promote the release of numerous autoantigens, leading to the development of autoantibodies against cytoplasmic and nuclear compounds such as ANA and dsDNA [40], especially if production of these autoantibodies is no longer suppressed by the action of infliximab on the suppressor T cell population. This apoptotic process might not occur in organ-specific cells because these cells, namely thyrocytes, do not harbor TNF-α receptor, thus shedding some light on the findings concerning the absence of organ-specific autoantibodies associated with autoimmune vasculitis, hepatitis or endocrine diseases.

Like Charles and colleagues [13], we demonstrated that most of the anti-dsDNA autoantibodies detected during the treatment of RA with infliximab were of IgM isotype. Furthermore, we showed that most of the detected aPL autoantibodies were also of IgM isotype. The role of these IgM in the development of autoimmune diseases remains to be elucidated. Natural autoreactive IgM autoantibodies might suppress autoimmunity by inducing B cell tolerance and thus by participating in the negative selection of autoreactive B cells. The larger pool of autoantibodies of IgM isotype observed during infliximab treatment might be the consequence of a higher production of natural autoreactive IgM, but might also be an induced population that can further switch to IgG with a well-known pathogenic effect. A high frequency of IgM might also result from TNF blockade, as it was demonstrated in a murine model of collagen-induced arthritis that anti-TNF-α monoclonal antibodies reduce isotype switching to IgG in the local draining lymph node [41].

## Conclusion

Our results show that infliximab induces ANA, anti-dsDNA and aPL autoantibodies at various times after the start of treatment. However, it seems that the development of such autoantibodies is not predictive of the development of SLE-like syndrome, because during the 2-year follow-up of infliximab therapy no APL syndrome or SLE syndrome appeared. Nevertheless, these findings do not exclude the possibility that such pathology might develop after a longer period of infliximab treatment. They underline the need to monitor the humoral response, namely autoantibodies and clinical manifestations, in patients treated with infliximab over a longer period.

## Competing interests

None declared.

## Abbreviations

ACL = anticardiolipin; ADA = anti-adrenal autoantibodies; AMA = anti-mitochondrial autoantibodies; ANA = antinuclear autoantibodies; ANCA = anti-neutrophil cytoplasmic autoantibodies; aPL = antiphospholipid; AS = ankylosing spondylitis; dsDNA = double-stranded DNA; ELISA = enzyme-linked immunosorbent assay; ENA = anti-extractible nuclear antigen; β_2_GPI = β_2_-glycoprotein I autoantibodies; GPL = G phospholipid; IIF = indirect immunofluorescence; LKM = anti-liver kidney microsomes; MPL = M phospholipid; RA = rheumatoid arthritis; SLE = systemic lupus erythematosus; SMA = anti-smooth muscle; TG = anti-thyroglobulin; TNF-α = tumor necrosis factor α; TPO = thyroid peroxidase.

## Figures and Tables

**Table 1:** Clinical characteristics of patients

	Control	Rheumatoid arthritis	Ankylosing spondylitis
Number of patients	30	24	15
Mean age, years (range)	63 (30–83)	56 (26–77)	41 (26–57)
Number of women (%)	23 (76.7)	16 (66.7)	4 (26.7)
Disease duration, years (range)	7.4 (1–22)	12 (3–32)	17 (6–30)
Infliximab treatment (mg/kg)	0	3	5
Concomitant medication			
Number of patients with			
NSAID	22	10	5
Corticosteroids	19	19	8
Methotrexate	30	24	6
Side effects			
Number of patients with			
Allergy		3 M^a^, 1 S^a^	1 S^a^
Infections		2 M^a^, 2 S^a^	2 M^a^, 1 S^a^
Other (amyloidosis)		0	3 M^a^
Inefficacy of treatment			
Number of patients		2	2
		Months 8 and 10	Months 5 and 7

AS, ankylosing spondylitis; NSAID, non-steroidal anti-inflammatory drugs; RA, rheumatoid arthritis.

^a^Significant side effects and inefficacy that could lead to infliximab discontinuation. If side effects were severe (S), infliximab was stopped; thus nine patients discontinued infliximab treatment before the end of the study, between 3 and 18 months in 5 RA and 2 AS patients. If moderate (M), infliximab was continued. The severe infections were pulmonary tuberculosis (RA), septic pericarditis (AS) and *Streptococcus bovis *endocarditis (RA). Two severe anaphylactic reactions during infliximab infusion (1 RA, 1 AS) led to discontinuation of treatment and in one case (RA) required resuscitation. Three patients with severe long-standing AS were suspected of amyloidosis at the start of infliximab treatment because of nephritic proteinuria. The diagnosis was confirmed by renal biopsy and the infusion carried out.

**Table 2:** Detection of ANA and anti-dsDNA autoantibodies during infliximab treatment

	Number of positive sera	ANA	Anti-dsDNA autoantibodies
Infliximab RA (*n *= 24)	***n***_**0**_	**9**	**1**
	*n*_i12_	12	10
	*n*_i24_	13	13
	***n***_**t**_	**22**	**14**
Infliximab AS (*n *= 15)	***n***_**0**_	**2**	**2**
	*n*_i12_	8	3
	*n*_i24_	11	4
	***n***_**t**_	**13**	**6**
Control RA (*n *= 30)	***n***_**0**_	**5**	**0**
	*n*_i12_	4	2
	***n***_**t**_	**9**	**2**

ANA, antinuclear autoantibodies; anti-dsDNA, anti-double-stranded DNA autoantibodies; *n*_0_, number of positive sera before treatment; *n*_i12_, number of positive sera-induced autoantibodies after 12 months of treatment; *n*_i24_, number of positive sera-induced autoantibodies after 24 months of treatment; *n*_t_, number of positive sera before treatment plus number of induced autoantibodies during infliximab treatment.

**Table 3:** Detection of ACL and anti-β_2_GPI autoantibody-positive sera during infliximab treatment

	Number of positive sera	Anti-β_2_GPI autoantibodies^a^		aCL autoantibodies^b^	
			
		IgG	IgM	IgG	IgM
Infliximab, RA (*n *= 24)	*n*_0_	0	0	0	0
	*n*_t_	2	1^c^	0	5
Infliximab, AS (*n *= 15)	*n*_0_	0	0	0	0
	*n*_t_	0	2	3	1
Control, RA (*n *= 30)	*n*_0_	0	0	0	0
	*n*_t_	0	0	0	0

Anti-β_2_GPI, anti-β_2_-glycoprotein I autoantibodies; aCL, anticardiolipin autoantibodies; *n*_0_, number of positive sera before treatment; *n*_t_, number of positive sera before treatment plus number of induced autoantibodies during infliximab treatment.

^a^Anti-β_2_GPI with one high titer (5.4 AU/ml [IgG], 5 AU/ml [IgM]) and one moderate titer (2.1 AU/ml [IgG]), in 2 of 15 AS with two high titers (8.7 AU/ml [IgG], 3.4 AU/ml [IgM]).

^b^Low, moderate and high titers were observed in two patients (15.1, 23 G phospholipid), in six patients (22.4, 22.8, 15.4, 21, 22.1 M phospholipid, 27 G phospholipid), and in one patient (41.5 M phospholipid) respectively.

^c^Both IgG and IgM.
